# Therapeutic analysis of laser moxibustion for different KL graded knee osteoarthritis

**DOI:** 10.1097/MD.0000000000038567

**Published:** 2024-06-21

**Authors:** Yuming Yan, Lin Lin, Ke Cheng, Haiping Deng, Meng Qin, Xueyong Shen, Ling Zhao

**Affiliations:** a School of Acupuncture-Moxibustion and Tuina, Shanghai University of Traditional Chinese Medicine, Shanghai, China; b School of Shanghai Research Center of Acupuncture and Meridian, Shanghai, China.

**Keywords:** 10.6 μm laser moxibustion, knee osteoarthritis, pain, traditional Chinese medicine

## Abstract

**Background:**

Our previous studies showed that laser moxibustion may be effective in alleviating the symptoms of knee osteoarthritis. However, the therapeutic effect in patients with different Kellgren-Lawrence (KL) grades is still unclear. We aimed to compare the efficacy of laser moxibustion in the treatment of knee osteoarthritis with different KL grades.

**Methods:**

A total of 392 symptomatic KOA patients with different KL grades were randomly assigned to the laser treatment or sham laser control group (1:1). The patients received laser moxibustion treatment or sham treatment 3 times a week for 4 weeks. Outcomes were measured using the Western Ontario and McMaster Universities Arthritis Index (WOMAC) scores and Visual Analog Scale (VAS) scores, and the primary outcome measurement was the change in WOMAC pain scores from baseline to week 4.

**Results:**

Among 392 randomized participants, 364 (92.86%) completed the trial. Participants with KL grades 2, 3, and 4 had significantly higher pain, functional, and total WOMAC scores than those with KL grade 1. Spearman correlation test results showed a positive correlation between KL grade and WOMAC pain, function, stiffness scores, and WOMAC total scores. That is, the higher the KL grade, the higher the WOMAC pain, function, stiffness, and WOMAC total scores. After 4 weeks of treatment, patients with KL grades 2 and 3 had significantly higher improvement scores in pain, function, and total scores than those with KL grade 1, whereas those with KL grade 2 had significantly higher improvement scores in stiffness than those with KL grade 1. Patients with KL grade 4 showed no significant effects after laser moxibustion treatment.

**Conclusion:**

Laser moxibustion is effective for pain reduction and functional improvement in the treatment of KOA with KL grades 2 and 3.

**Key points**

- By analyzing preliminary clinical trial data, the therapeutic effect of 10.6 µm laser moxibustion on knee osteoarthritis with different KL grades was observed, and it was confirmed that KL grades were positively correlated with WOMAC pain, function, stiffness scores, and WOMAC total scores.
- After 4 weeks of treatment, patients with KL grades 2 and 3 had significantly higher improvement scores in pain, function, and total scores than those with KL grade 1, whereas those with KL grade 2 had significantly higher improvement scores in stiffness than those with KL grade 1.
- The results suggest that laser moxibustion has different therapeutic effects on different grades of knee osteoarthritis, thus providing evidence for the dose-effect study of laser moxibustion on knee osteoarthritis in the future.

## 1. Introduction

Osteoarthritis (OA) is the most common form of arthritis and the leading cause of disability among older adults. The knee is the joint most commonly affected by osteoarthritis.^[1]^ Osteoarthritis affects an estimated 240 million people worldwide,33% of individuals older than 75 years have symptomatic and radiographic knee osteoarthritis (KOA).^[2]^ The prevalence of KOA in China is nearly 14.6%^[3]^ and the weighted prevalence of radiographic KOA among adults age ≥50 years was 35.1% from the Korean.^[4]^ Analyses from The Chingford Study found that the cumulative 5-year incidence of “typical” radiographic KOA among women age 45 to 64 years was 17.6%, and the incidence of “accelerated” radiographic KOA was 3.7%.^[5]^ Conventional treatment of KOA mainly aims at alleviation of pain including pharmacological and non-pharmacological managements, such as nonsteroidal anti-inflammatory drugs and exercise therapy, weight management, biomechanical intervention, and so on.^[6–12]^ Nonsteroidal anti-inflammatory drugs are associated with a moderate effect on pain relief,^[6,7]^ however, evidence on their effectiveness is limited^[6–10]^ and it is not suitable for long-term use because of its side effects.^[9,10,13]^ According to the traditional Chinese medicine theory, joint pain is associated with coldness and dampness. Thermotherapy is often used to treat joint pain. Moxibustion, as an important means of warm and hot treatment in traditional Chinese medicine, has been proven to be effective and has fewer side effects in the treatment of KOA. However, moxibustion therapy produces heavy smoke with an unpleasant smell, and multiple studies have shown that moxibustion smoke is considered a biological hazard to health.^[14–16]^ In our previous randomized controlled clinical trial, we developed a laser moxibustion (LM) device of 10.6 µm wavelength of moxibustion without smoke and smell. Our previous small studies showed that laser moxibustion may be effective in alleviating the symptoms of KOA.^[17]^ However, the therapeutic effect in patients with different KL grades is still unclear. To overcome the limitations of the previous article, 392 patients were analyzed to compare the efficacy of laser moxibustion in the treatment of KOA with different KL grades.

## 2. Methods

### 2.1. Study design

This was a secondary analysis of the results of a previous multi-site randomized double-blind controlled trial (ISRCTN registry trial identifier:15030019).^[18]^ The trial protocol adhered to CONSORT guidelines. It was conducted in the outpatient clinics of 6 hospitals in Shanghai, China, and was approved by the Institutional Review Board at each site. A total of 392 patients with different KL grades were included in this secondary analysis, and the number of patients with different KL grades was confirmed to investigate the effects of laser moxibustion. All patients provided written informed consent before the start of the study. The study for each patient included 2 weeks of baseline assessment and 4 weeks of treatment.

### 2.2. Participants

In our secondary analysis, we included 392 participants who had been diagnosed with KOA and reported experiencing moderate or more severe clinically significant knee pain on most days over the past month. Among these participants, baseline radiographs revealed that 267 had KL grade 1, 240 had KL grade 2, 378 had KL grade 3, and 47 had KL grade 4 out of the total 392 patients.

### 2.3. Randomization and interventions

A total of 392 eligible participants were randomly assigned to either the laser treatment or the sham laser control group. The treatments lasted 20 minutes in each session and were performed 3 times a week for 4 weeks. Selecting ST35 (Du bi) and Ashi point (tender point) for the treatment.^[19]^ The laser moxibustion devices’s wavelength of laser irradiation was 10.6 µm, and the output power was adjusted in the range of 160 to 180 mW, energy density ranged from 61.2 to 68.8 J/cm^2^ for one treatment.

### 2.4. Outcome assessment

Patients were assessed at baseline and week 4. The primary outcome was the change in Western Ontario and McMaster Universities Osteoarthritis Index (WOMAC)^[20]^ pain scores from baseline to 4 weeks. Secondary outcomes included changes in WOMAC scores at week 4 and health-related quality of life (as measured by the 36-Item Short Form Health Survey [SF-36]^[21]^). Adverse events, whether related to treatment or not, reported by participants and practitioners will be documented.

### 2.5. Statistical methods

SPSS software (version 21.0) was used for the data analysis. All numerical data are expressed as mean ± standard deviation. Comparison of WOMAC and SF-36 scores in different KL grades was analyzed with One-Way ANOVA or Kruskal Wallis test. Least significant difference (LSD) method was used for multiple comparison. Spearman nonparametric correlation test was used for statistical analysis. Differences were considered statistically significant at *P* value <.05.

## 3. Results

### 3.1. Participant characteristics

After initial screening, 392 patients were randomly assigned to either the LM group (n = 201) or the sham LM control group (n = 191). Three hundred sixty-four patients (92.86%) completed the study and available for analysis.193 patients of laser moxibution group and 177 patients of sham laser control group completed all 12 sessions of therapy. No additional missing data other than those withdrawn from the study. Missing data of withdrawn participants were replaced with the data of last observation-carried-forward. Baseline characteristics were similar between the groups. Most study patients were women (75%). No significant difference was found between the 2 groups in age, sex, disease course, medication use, severity of disease, WOMAC scores for knee pain or physical function, and cytokine level. This result suggests that the 2 groups were comparable.

### 3.2. Outcomes

**Figure 1.**
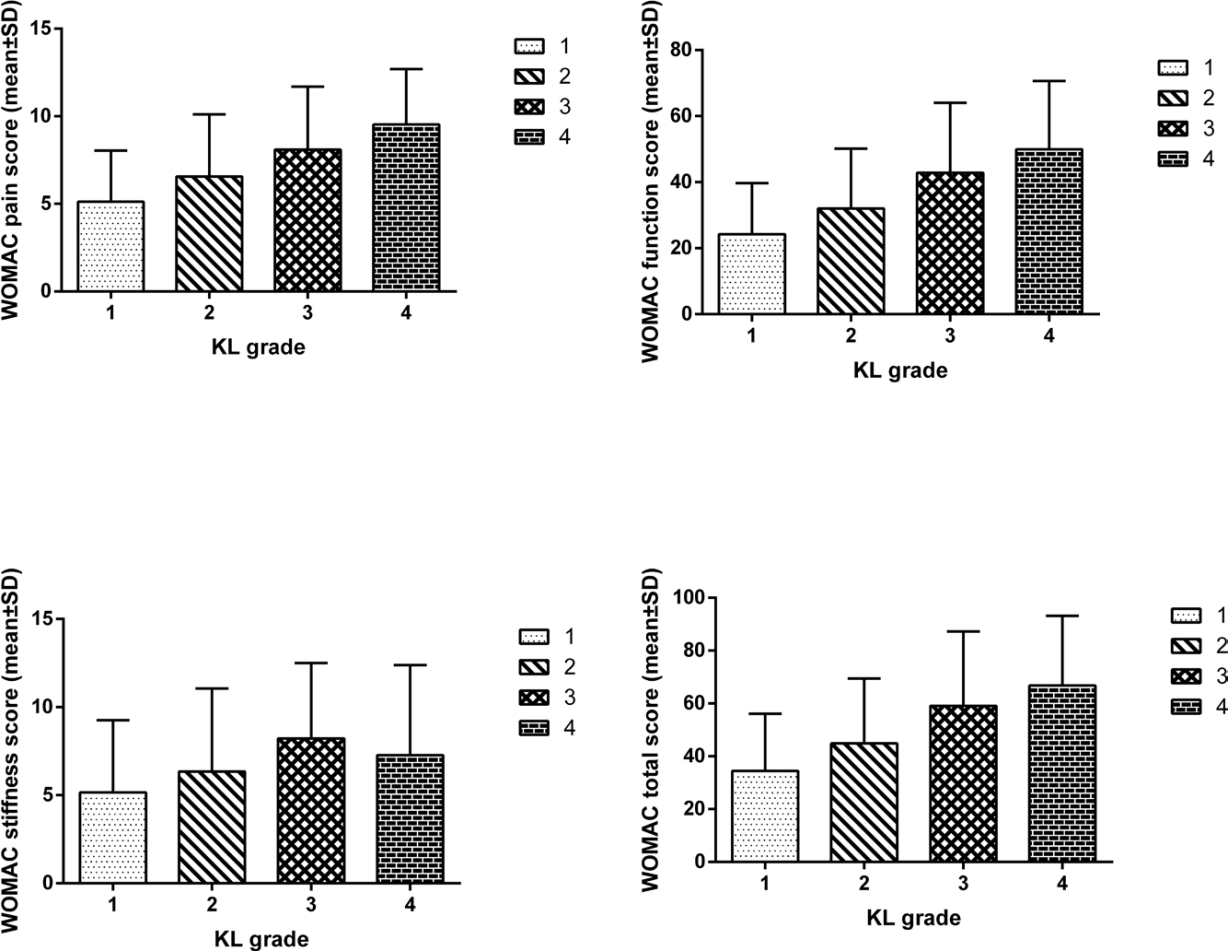
WOMAC scores of various dimensions of subjects with different KL grades before treatment.

Before treatment, there were statistically significant differences in WOMAC pain, function, stiffness, and WOMAC total scores among subjects with different KL grades. According to multiple comparison (LSD) statistics, the pain, function, and WOMAC total scores of subjects with KL grades 2, 3, and 4 were significantly higher than those with KL grade 1. The pain, function, and WOMAC total scores of subjects with KL grades 3 and 4 were significantly higher than those with KL grade 2, and the stiffness scores of subjects with KL grade 3 were significantly higher than those with KL grades 1 and 2. The scores of KL grade 4 were higher than those of the other 3 grades, except for the stiffness scores (Fig. 1). Spearman correlation test showed that the KL rating was positively correlated with WOMAC pain, function and stiffness scores, and WOMAC total scores. The higher the KL rating, the higher the WOMAC pain, function, and stiffness scores and the WOMAC total scores.

**Figure 2.**
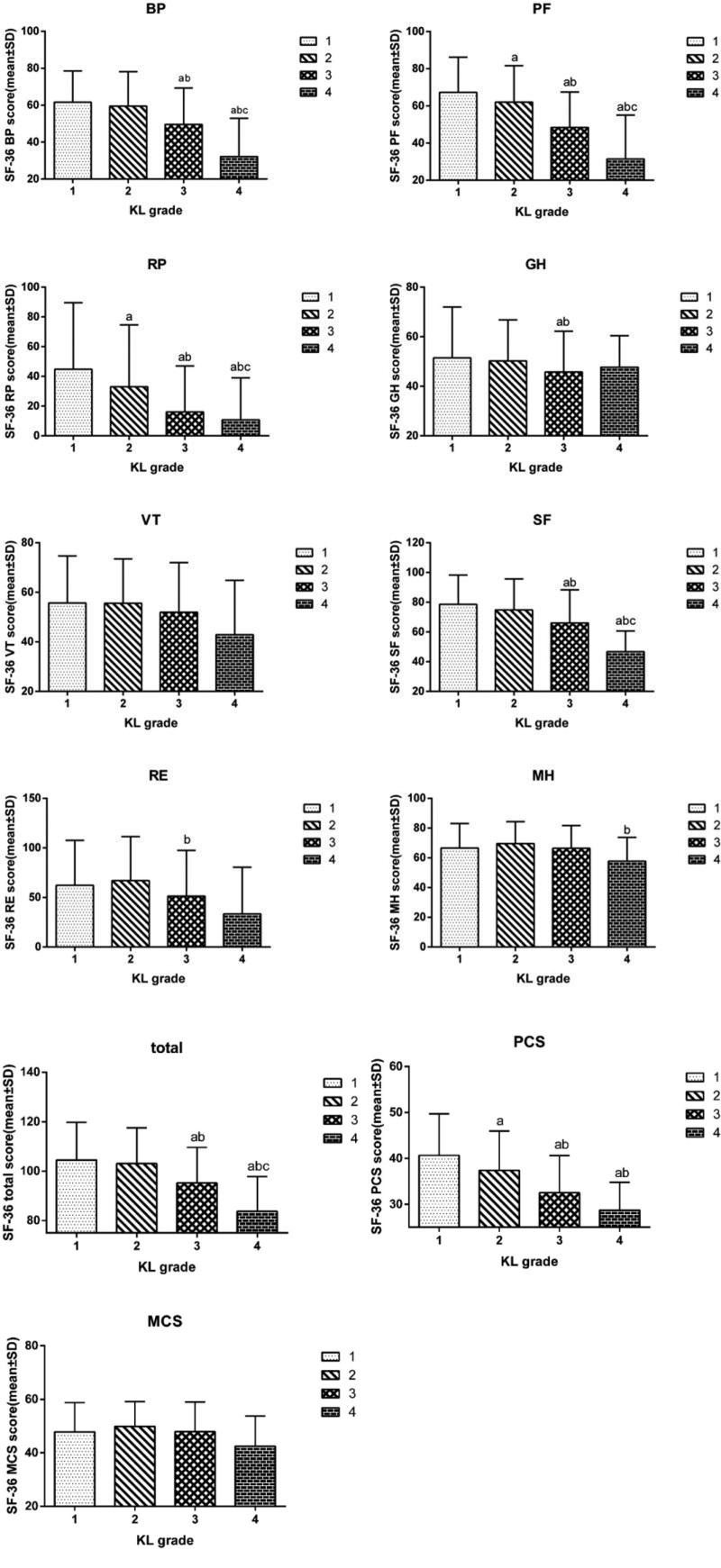
SF-36 scores of subjects with different KL grades before treatment.

Before treatment, the scores of bodily pain, physical functioning, role-physical, social functioning (SF), role-emotional, general health, physical component summary (PCS) on the SF-36 scale of subjects with different KL grades were significantly different. Spearman correlation test showed that KL grading was negatively correlated with bodily pain, physical functioning, role-physical, general health, SF, and PCS. That is, the higher the KL grade, the lower the SF total scores and the lower the PCS of physical health total scores, but there was no significant correlation between KL grades and mental health (Fig. 2).

**Figure 3.**
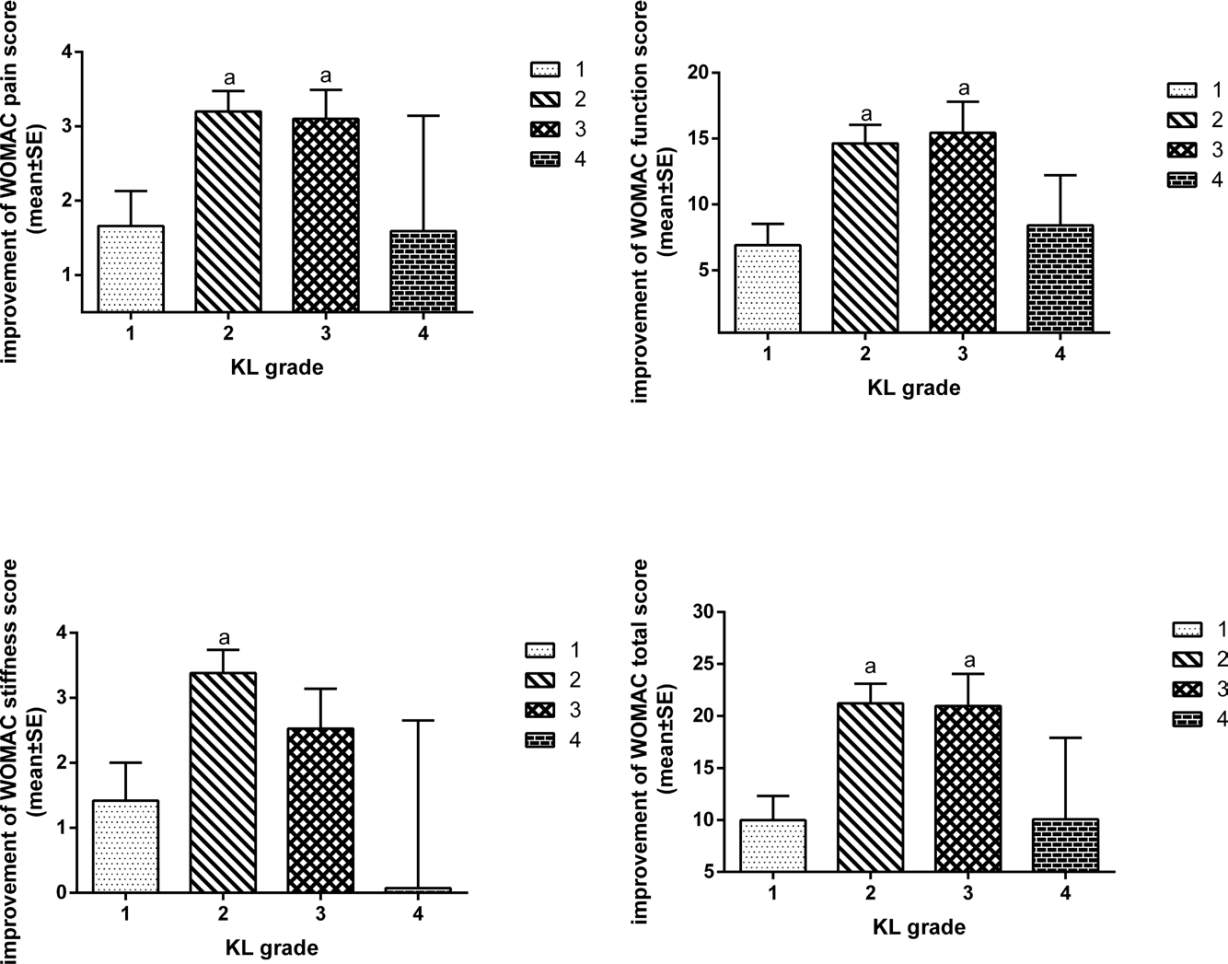
WOMAC improvement in all dimensions of subjects with different KL grades in the laser moxibustion group after treatment.

After 4 weeks of treatment, pain, function, stiffness, and WOMAC total scores were significantly lower in subjects with KL grades 1 and 3 than before treatment, as analyzed by paired *t*-tests (*P* = .001, .021, .001, .001). Pain, function, stiffness, and WOMAC total scores were significantly lower in subjects with KL grade 2 than in those before treatment (*P* < .001). There was no difference in pain, function, stiffness, and WOMAC total scores between subjects with KL grade 4 and those before treatment(*P* = .415, .982, .157, .329).There were significant differences in pain, function, stiffness, and WOMAC scores among the subjects with different KL grades (*P* < .05). The results of multiple comparisons (LSD) showed that the pain, function, stiffness, and overall improvement scores of KL grades 2 and 3 were significantly higher than those of KL grades 1 and 4 (Table 1 and Fig. 3).

**Table 1:** WOMAC improvement in subjects with different KL grades in laser moxibustion group after treatment.

WOMAC	Kellgren and Lawrence grade				F	*P*
	1 (n = 33)	2 (n = 122)	3 (n = 43)	4 (n = 3)		
Pain	1.66 ± 2.69†	3.20 ± 3.05*^,^†	3.10 ± 2.56*^,^†	1.59 ± 2.69	2.679	.048
Function	6.90 ± 9.23†	14.63 ± 15.56*^,^†	15.43 ± 15.56*^,^†	8.41 ± 6.59	2.843	.039
Stiffness	1.42 ± 3.36†	3.38 ± 3.96*^,^†	2.53 ± 3.98†	0.07 ± 4.47	2.845	.039
Total	9.98 ± 13.40†	21.21 ± 21.03*^,^†	20.96 ± 20.29*^,^†	10.07 ± 13.62	3.141	.026

KL = Kellgren-Lawrence, WOMAC = Western Ontario and McMaster Universities Arthritis Index.

* Compare with grade 1: *P* < 0.05.

† Compare with before the treatment *P* < .05.

**Figure 4.**
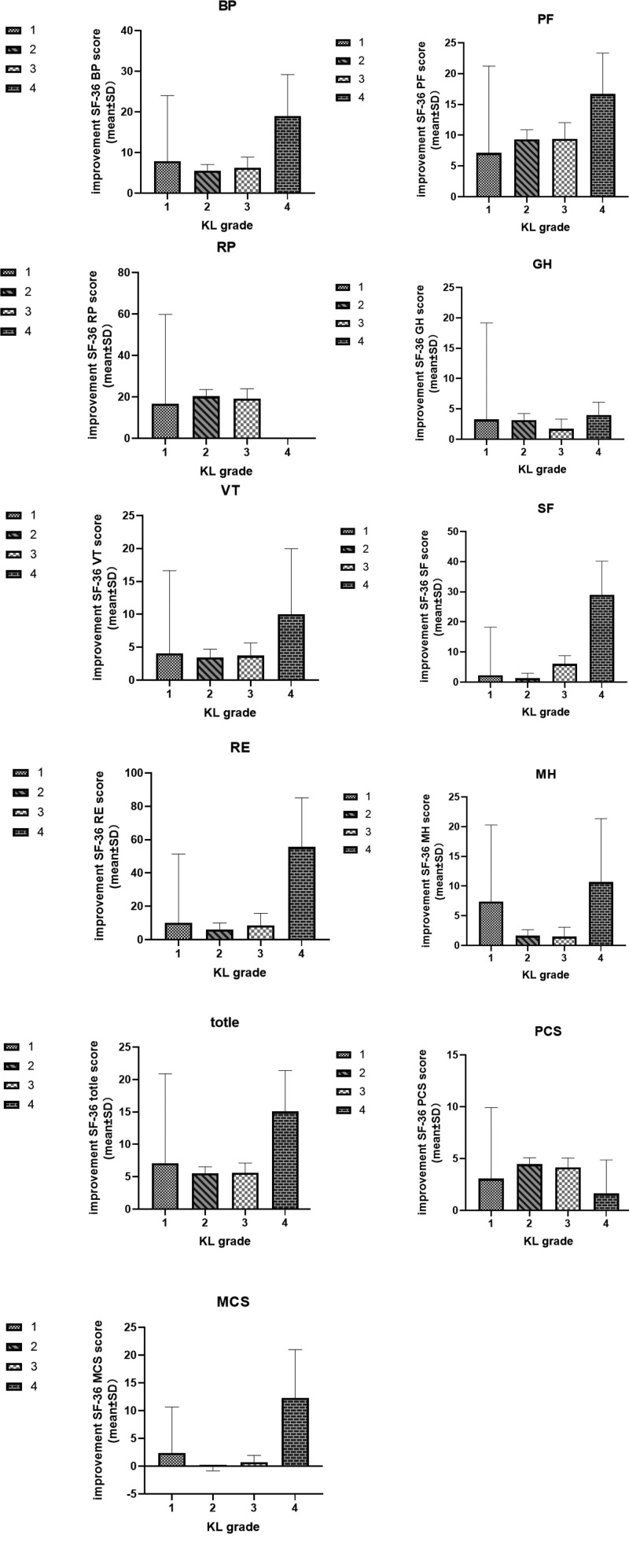
SF-36 scores of subjects with different KL grades after treatment.

After treatment, the SF total scores and overall physical health PCS scores of subjects with KL grades 1, 2, and 3 in the laser moxibustion group were significantly lower than those in the pretreatment *(P* < .05). There was no significant difference between the total SF and PCS scores of subjects with KL grade 4 compared with those before treatment (*P* > .05). There was no significant difference in the overall mental health rating of all subjects compared with that before treatment (*P* > .05) (Fig. 4). A comparison of the scores of the other dimensions with those before treatment is shown in Table 2 and Figure 4.

**Table 2:** The improvement of SF-36 of subjects with different KL grades in laser moxibustion group after treatment.

Kellgren and Lawrence grade	n	BP	PF	RP	GH	VT	SF	RE	MH	SF totle	PCS	MCS
1	33	7.88 ± 16.14*	7.12 ± 14.09*	16.67 ± 43.15*	3.27 ± 15.89*	4.09 ± 12.53*	2.24 ± 16.01*	10.09 ± 41.25*	7.39 ± 12.89	7.08 ± 13.82*	3.07 ± 6.85*	2.38 ± 8.29
2	122	5.55 ± 16.66*	9.34 ± 17.11*	20.29 ± 35.98*	3.16 ± 11.69*	3.48 ± 13.68*	1.26 ± 18.90	6.02 ± 44.54	1.64 ± 11.31	5.52 ± 11.08*	4.46 ± 6.94*	-0.15 ± 7.52
3	43	6.26 ± 17.19*	9.41 ± 17.19*	19.19 ± 30.78*	1.72 ± 10.42	3.72 ± 12.73	6.07 ± 17.90*	8.51 ± 47.84	1.49 ± 10.29	5.60 ± 10.02*	4.15 ± 5.89*	0.75 ± 7.89
4	3	19.00 ± 17.69	16.67 ± 11.55	0.00 ± 0.00	4.00 ± 3.61	10.00 ± 17.32	29.00 ± 19.31	55.67 ± 50.95	10.67 ± 18.48	15.07 ± 10.95	1.63 ± 5.61	12.31 ± 15.02
F		0.545	0.569	0.400	0.093	0.201	0.107	0.079	7.545	1.452	0.788	0.490
*P*		.460	.451	.527	.761	.654	.744	.778	.006	.228	.375	.228

BP = body pain, GH = general health, KL = Kellgren–Lawrence, MCS = mental component summary, MH = mental health, PCS = physical component summary, PF = physical functioning, RE = role-emotional, RP = role-physical, SF = social functioning, VT = vitality.

* Compare with before the treatment: *P* < 0.05.

### 3.3. Adverse events

Of the 391 participants, adverse effects were reported by 30 individuals (7.65%) in the active laser moxibustion group and 6 individuals (3.14%) in the sham laser group. The most frequently reported adverse effect was skin rash, with 21 cases among those who received active laser moxibustion; all individuals with a skin rash fully recovered within 3 days.

## 4. Discussion

The KL grading method is widely used to evaluate KOA degree. It has been found that KL level was positively correlated with the severity and pain of KOA; the higher the KL grade of the patient, the more severe the KOA, the more intense the pain will be^[22]^ and the presence of depressive symptoms is associated with an increased risk of severe knee pain.^[23,24]^ Studies have shown that radiographic grading with the Kellgren–Lawrence scale revealed a significant relationship to the joint space width and joint space, the joint space width decreased with increasing Kellgren–Lawrence grade,^[25]^ the range of joint motion was significantly reduced with the increase in KL grading.^[26]^ The severity of KL grade was associated with a variety of cytokines and telomere size,^[27–29]^ these cytokines are signaling molecules involved in inflammation and immune responses, telomere size decreases with the increase in KL grading, shorter telomeres indicate premature aging, which aggravates the aging of chondrocytes and degenerative changes in joints.^[30]^

Our study included 392 patients with osteoarthritis of the knee aged 50 to 75 years. We found that the severity of the Kellgren–Lawrence (KL) grading was positively correlated with Western Ontario and McMaster Universities Osteoarthritis Index (WOMAC) pain, function, and stiffness scores, as well as the total scores on the WOMAC scale. In other words, the higher the severity of KL, the higher the WOMAC pain, function, and stiffness scores, as well as the total scores on the WOMAC scale. It has been found^[23]^ that the frequency and severity of pain in patients with osteoarthritis of the knee are most closely related to joint space narrowing.

Few studies have assessed the relationship between quality of life and KL severity in patients with KOA. In this study, we found that the higher the patients’ KL grading, the lower the SF-36 total scores, and the worse their quality of life. In terms of the total physical health scores (PCS), and mental component summary (MCS), the total PCS scores was negatively correlated with KL severity, but the MCS was not correlated with it. Therefore, KOA mainly affects patients’ quality of life related to physical health. We also calculated the correlation between WOMAC pain and SF-36 mental health total scores MCS in 392 subjects and found no correlation between the 2 (*R* = 0.004, *P* = .931). Therefore, we believe that osteoarthritis of the knee reduces the quality of life of patients, especially affecting their physical health, and has a nonsignificant effect on mental health. Marcos Edgar Fernandez-Cuadros et al^[31]^ believed that KOA can have a negative impact on the mental health of patients, especially women and the elderly.^[32]^ Knee osteoarthritis can restrict all aspects of life, and the elderly may suffer from increasingly serious complications,^[33]^ which will greatly affect the mental health of patients. The mental health of patients after total knee arthroplasty is significantly improved compared with that before treatment, which may be related to the fact that the condition of patients in need of total knee replacement is usually more serious, and the impact on mental health is greater. In our study, since we included patients who have no history or plan of knee/hip replacement surgery, their condition may not be so serious, and the impact on mental health is less. The results of the study by Kim et al^[34]^ suggest that the assessment and treatment of depression should be included in orthopedic care for patients with KOA to evaluate the mental health of patients with KOA.

After 12 laser moxibustion treatments over 4 weeks, an analysis of 200 subjects with different KL grades in the true laser moxibustion group found that WOMAC pain, function, stiffness, and total scores significantly improved after treatment compared to before treatment in all 3 classes, except those with a KL grade of 4. Regarding quality of life, the SF scale total scores as well as total health scores (PCS) of subjects with a KL grading of 1 to 3 improved significantly from before treatment, and there was no difference from before treatment in subjects with a KL grading of 4. There was no statistically significant difference in the total MCS of all graded patients compared to before treatment. From the results, it can be seen that laser moxibustion treatment was not effective for subjects with KL grading of 4. Therefore, this study provides a reference for clinicians and patients with KOA, indicating that laser moxibustion or moxibustion treatment is not preferentially recommended for subjects with KL grade 4. Laser moxibustion treatment can be chosen to improve symptoms in all other 3 grades, especially in grades 2 and 3, that is, moderate.

## 5. Limitations

There are still some limitations of this study, such as the lack of measurement of specialized psychiatric depression-related scales and posttreatment imaging of the patients. In our study, we included only 7 patients in KL 4 grade group, a larger sample study should be conducted to make the observations more persuasive in the future.

## 6. Conclusion

Laser moxibustion is effective for pain reduction and functional improvement in treating KOA with KL grades 2 and 3.

## Acknowledgments

The authors would like to thank the patients of this study for their cooperation and commitment.

## Author contributions

**Data curation:** Ke Cheng, Haiping Deng, Meng Qin.

**Writing – original draft:** Yuming Yan, Lin Lin.

**Writing – review & editing:** Xueyong Shen, Ling Zhao.
